# Quasi-type II CuInS_2_/CdS core/shell quantum dots†

**DOI:** 10.1039/c5sc03715h

**Published:** 2015-11-12

**Authors:** Kaifeng Wu, Guijie Liang, Degui Kong, Jinquan Chen, Zheyuan Chen, Xinhe Shan, James R. McBride, Tianquan Lian

**Affiliations:** a Department of Chemistry, Emory University 1515 Dickey Drive, NE Atlanta Georgia 30322 USA tlian@emory.edu; b Hubei Key Laboratory of Low Dimensional Optoelectronic Materials and Devices, Hubei University of Arts and Science Xiangyang 441053 Hubei Province P. R. China; c College of Electronic Engineering, Heilongjiang University Harbin 150080 P. R. China; d Department of Chemistry, The Vanderbilt Institute of Nanoscale Science and Engineering, Vanderbilt University Nashville TN 37235 USA

## Abstract

Ternary chalcopyrite CuInS_2_ quantum dots (QDs) have been extensively studied in recent years as an alternative to conventional QDs for solar energy conversion applications. However, compared with the well-established photophysics in prototypical CdSe QDs, much less is known about the excited properties of CuInS_2_ QDs. In this work, using ultrafast spectroscopy, we showed that both conduction band (CB) edge electrons and copper vacancy (V_Cu_) localized holes were susceptible to surface trappings in CuInS_2_ QDs. These trap states could be effectively passivated by forming quasi-type II CuInS_2_/CdS core/shell QDs, leading to a single-exciton (with electrons delocalized among CuInS_2_/CdS CB and holes localized in V_Cu_) half lifetime of as long as 450 ns. Because of reduced electron–hole overlap in quasi-type II QDs, Auger recombination of multiple excitons was also suppressed and the bi-exciton lifetime was prolonged to 42 ps in CuInS_2_/CdS QDs from 10 ps in CuInS_2_ QDs. These demonstrated advantages, including passivated trap states, long single and multiple exciton lifetimes, suggest that quasi-type II CuInS_2_/CdS QDs are promising materials for photovoltaic and photocatalytic applications.

## Abstract 2

Both single and multiple exciton lifetimes are significantly extended in quasi-type II CuInS_2_/CdS core/shell QDs.

## Graphical abstract

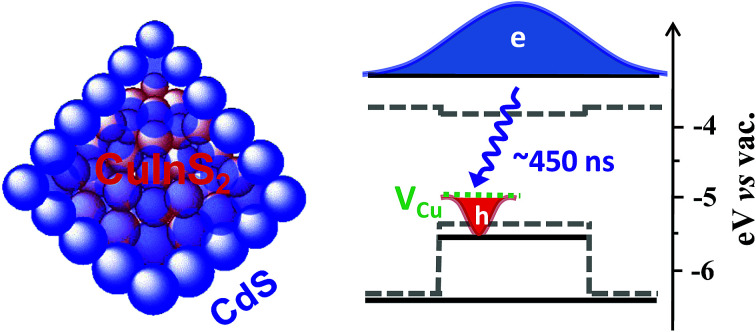

## Introduction

Ternary chalcopyrite CuInS_2_ has a direct bulk bandgap of 1.5 eV,^1^ which matches well with the solar spectrum for photovoltaic or photocatalytic applications.^2–6^ The absorption and emission of colloidal CuInS_2_ quantum dots (QDs) can cover most of the visible and near IR range by tuning their sizes.^7–9^ Moreover, recently developed non-injection and high chemical yield colloidal syntheses of mono-dispersed CuInS_2_ QDs are promising for scaled-up production in industry.^4,9^ For these reasons, CuInS_2_ QDs have been extensively studied in recent years as an alternative to conventional II–VI CdX (X = S, Se, Te) QDs. CuInS_2_ QDs sensitized solar cells (QDSSCs) with power conversion efficiencies higher than 5% have been reported,^3,6,10^ exceeding those of CdSe QDSSCs.^11^ The radiative lifetime in CuInS_2_ QDs is found to be surprisingly long (∼300 ns),^4,12–17^ which was generally attributed to slow recombination between the conduction band (CB) edge electrons and holes trapped in defect states likely associated with Cu vacancies (V_Cu_).^4,17,18^ Additionally, there have been reports about other defects,^9,19^ such as sulfur vacancy (V_S_), indium vacancy (V_In_), interstitial copper (Cu_i_), copper site substituted by indium (In_Cu_), and indium site substituted by copper (Cu_In_). These off-stoichiometry defects, typical for CuInS_2_ because of its ternary chemical composition,^19^ make its excited state dynamics more complicated and much less well understood than prototypical CdSe QDs.^20–23^

**Scheme 1:**
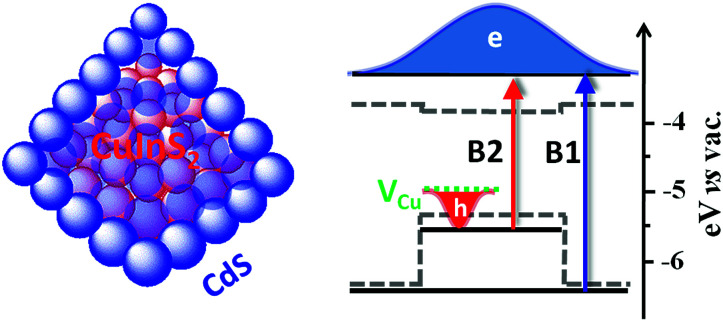
(Left) schematic structure and (right) quasi-type II band alignment in CuInS_2_/CdS core/shell QDs. Bulk band edges of CuInS_2_ and CdS (gray dashed lines), electron and hole energy levels in the core and shell (black solid lines), hole trapping levels associated with Cu vacancy (V_Cu_, green dashed line), lowest energy transitions in the CdS (B1) and CuInS_2_ (B2). In the lowest excited state, electron and hole wavefunctions are delocalized among core and shell and localized in the core (V_Cu_), respectively.

In addition to off-stoichiometry defects in the bulk, surface states are also found to play a significant role in carrier trapping for CuInS_2_ QDs,^4,16,24^ which reduces charge separation efficiencies in related photovoltaic and photocatalytic devices. Coating QDs with another material to form core/shell hetero QDs has been an effective approach to mitigating surface trapping states.^25,26^ Furthermore, core/shell QDs can lead to new properties that cannot be achieved in single component QDs.^25,27–33^ Depending on the relative alignment of the CB and valence band (VB) positions of the core and shell materials, core/shell QDs can be type I (in which the CB and VB of the core are nested between those of the shell^25,29^), type II (when they are staggered with respect to each other^34,35^), or quasi-type II (when either their CB or VB band edge positions are similar^26,36,37^). For CuInS_2_ QDs, it has been demonstrated that surface trapping can be suppressed and therefore photoluminescence (PL) quantum yields (QYs) can be strongly enhanced by coating with ZnS or CdS shells.^4,16,24,38^ It is well known that CuInS_2_/ZnS QDs have type I band alignment where both the lowest energy electron and hole wavefunctions are confined in the core so that the effect of surface states is reduced. However, for photovoltaic and photocatalytic applications, (quasi-)type II QDs are more suitable because their (partially) separated electron and hole wavefunctions can significantly prolong single and multiple exciton lifetimes.^35,39,40^ The CB and VB offsets between CuInS_2_ and CdS are ∼0.05 eV and 0.95 eV (Scheme 1),^41^ respectively. In principle, these offsets allow the formation of quasi-type II core/shell QDs where holes are well confined in CuInS_2_ and electrons are delocalized among CuInS_2_ and CdS (Scheme 1).

In this work, we present the first direct evidence for quasi-type II band alignment in CuInS_2_/CdS core/shell QDs and demonstrate their advantages for light harvesting and charge separation through comparison with core-only CuInS_2_ QDs. We show that both CB edge electrons and V_Cu_ localized holes were susceptible to surface trapping in CuInS_2_ QDs and these traps can be efficiently suppressed in CuInS_2_/CdS QDs to achieve a long-lived single exciton state (with electrons delocalized among CuInS_2_/CdS CB and holes localized in V_Cu_) with a half lifetime as long as 450 ns. Bi-exciton lifetime was prolonged from 10 ps in CuInS_2_ QDs to 42 ps in CuInS_2_/CdS QDs due to reduced electron–hole interactions in such quasi-type II QDs.

## Sample preparation and characterizations

**Fig. 1:**
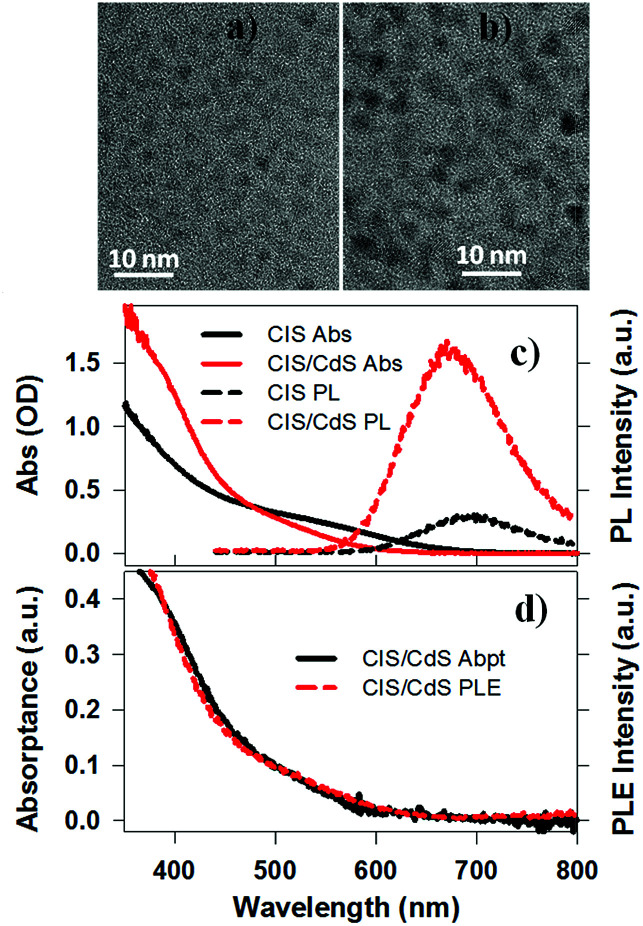
TEM images and absorption, PL and PLE spectra. Representative TEM images of (a) CuInS_2_ core, and (b) CuInS_2_/CdS core/shell QDs. (c) Absorption (solid lines) and photoluminescence (PL, dashed lines) spectra of CuInS_2_ core (black lines) and CuInS_2_/CdS core/shell QDs (red lines) dispersed in heptane. (d) Comparison between normalized absorptance (black solid line) and photoluminescence excitation (PLE, red dashed line) spectra of CuInS_2_/CdS core/shell QDs. They were normalized at the low energy side (500–600 nm).

CuInS_2_ (CIS) core and CuInS_2_/CdS (CIS/CdS) core/shell QDs were synthesized using a literature-reported method.^4^ Details can be found in the ESI.†Fig. 1a and b are representative Transmission Electron Microscopy (TEM) images of CIS core and CIS/CdS core/shell QDs, respectively. The CIS core exhibited typical tetrahedral shapes and the average size or edge length (standard deviations) was 3.34 (±0.95) nm. The size distribution histogram of CIS core is shown in Fig. S1a.† After CdS shell growth, the average size of core/shell QDs increased to 4.25 (±1.22) nm (Fig. S1b†). According to previous studies, CIS cores were possibly partially etched during shell growth^4^ and therefore we were not able to determine the core and shell sizes in our core/shell QDs. In the ESI,† we provided additional characterizations of the core/shell QDs using high-angle annular dark-field (HAADF) scanning TEM (Fig. S2†) and energy-dispersive X-ray spectroscopy (EDX, Fig. S3†). The EDX images showed spatially overlapped Cu, In, S, and Cd elements, consistent with uniform shell formation. Unfortunately, the differentiation between core and shell was still hindered by limited spatial resolution (∼0.5 nm) of EDX measurements, overlapping cadmium and indium X-ray peaks, and by the rate of electron beam-induced damage.

The UV-vis absorption spectra of CIS core and CIS/CdS core/shell QDs dispersed in heptane are shown in Fig. 1c. Unlike typical CdSe QDs, discrete exciton peaks were not observed for CIS. In ternary CIS QDs, there exist heterogeneous distributions of nanocrystal shapes, tetragonal lattice distortions, and compositional off-stoichiometry,^9^ all of which can contribute to inhomogeneous broadening of the exciton bands. As will be discussed below (Fig. 3), the lowest energy exciton peaks at ∼580 nm and ∼520 nm for CIS core and CIS/CdS core/shell QDs, respectively, can be clearly observed in transient absorption spectra. The blue shift of the exciton peak in CIS/CdS core/shell QDs could result from core etching during shell coating or alloying and cation exchange between core and shell, consistent with previous observations.^4,42,43^ If the core size and composition had remained unchanged during the shell growth, a red-shift should have been observed due to a reduction in the confinement energy.^25,26^ In addition to the blue-shift, the CIS/CdS QDs showed enhanced absorption in at <450 nm, which can be attributed to the absorption of the CdS shell.

Photoluminescence (PL) spectra of CIS and CIS/CdS QDs measured with 400 nm excitation are also shown in Fig. 1c. The CIS QDs showed a PL peak at ∼690 nm, which was red-shifted from the absorption peak (580 nm) by 0.34 eV. This Stokes-shift was almost an order of magnitude larger than in CdSe QDs and could be attributed to radiative recombination between CB electrons and V_Cu_ localized holes above the VB edge.^4,17,18^ The PL peak of CIS/CdS QDs (∼670 nm) was also blue-shifted from that of CIS QDs, consistent with absorption spectrum changes. As shown in Fig. 1d, the PL excitation (PLE) spectrum of CIS/CdS QDs (monitored the emission at 670 ± 1 nm), matched well with the absorptance spectrum (representing the percentage of absorbed photons), indicating that the PL quantum efficiency is independent on excitation wavelength. This result suggests that there was a negligible contribution of isolated CdS QDs and there was negligible exciton trapping at the CdS shell in CdSe/CdS QDs.^44,45^

### Ultrafast spectroscopic studies

#### Quasi-type II band alignment in CuInS_2_/CdS QDs

Transient absorption (TA) spectroscopy has been shown to be a powerful tool to investigate electronic structure (band alignment) and carrier dynamics in semiconductor nano heterostructures.^36,40,46–51^ The details of the pump-probe TA set-ups used in this study can be found in the ESI.† Briefly, a pump pulse with tunable excitation wavelengths and powers was used to excite the CIS or CIS/CdS QDs and the induced absorption changes, as a function of both wavelength and time, were recorded by a white light continuum (400–800 nm) probe pulse variably delayed (from fs to μs) with respect to the pump pulse.

**Fig. 2:**
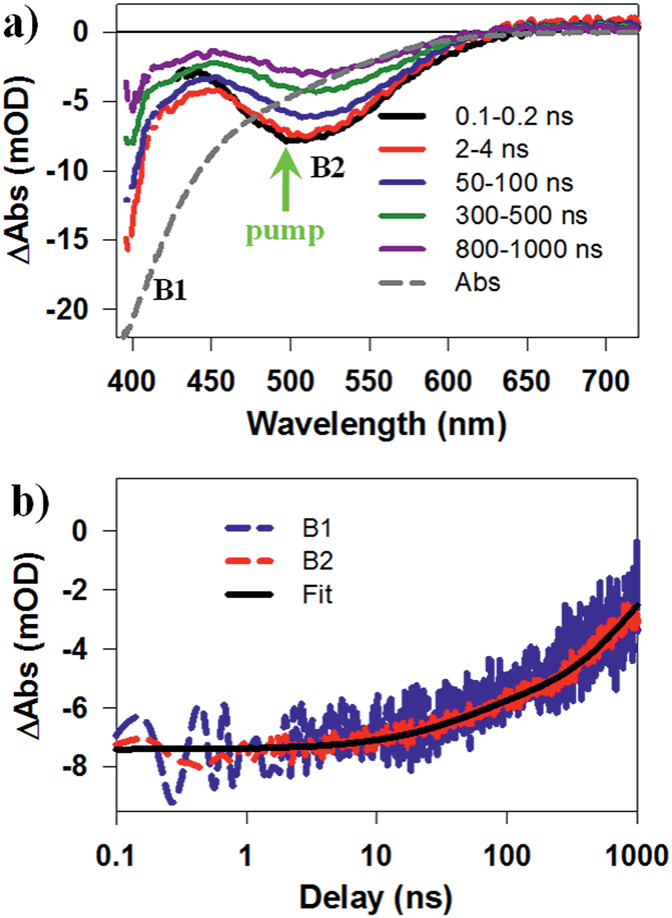
Transient Absorption (TA) spectra and kinetics of CuInS_2_/CdS core/shell QDs measured at 490 nm excitation. (a) TA spectra at indicated time delays (from 0.1 to 1000 ns). Static absorption spectrum (gray dashed line) is inverted and scaled for comparison. B1, B2 and excitation wavelength (green arrow) are labeled. (b) Comparison of TA kinetics of B1 (blue dashed line) and B2 (red dashed line) features and their multi-exponential fit (black solid line).

To identify the band alignment in CIS/CdS QDs, we used a pump wavelength at 490 nm, which selectively excited the CIS core but avoided excitation of CdS shell.^36,50^ The resultant TA spectra (Fig. 2a) showed a bleach of the lowest energy exciton band of CIS core at ∼520 nm (labelled as B2) and an additional bleach of the exciton band in CdS shell at ∼400 nm (labelled as B1). It has been well established experimentally that exciton bleach in CdS QDs is dominated by state filling of CB electron levels.^20,52^ Similar spectral assignment was found to be applicable to CIS QDs^4^ and we have confirmed this assignment by showing that the bleach features could be completely removed in the presence of electron acceptors (see Fig. S10, ESI† for details). As shown in Fig. 2b, the kinetics of B1 and B2 match well with each other, showing a half-life of ∼600 ns. The fitting parameters are listed in Table S1.† The simultaneous formation of long-lived B1 and B2 bleaches with the same decay kinetics after selective excitation of B2 can only be explained by a quasi-type II band alignment indicated in Scheme 1: the lowest electron level is degenerate among the CIS core and CdS shell. The energy difference between the B1 and B2 transitions (∼0.62 eV) reflects the large VB edge offset in these materials.

#### Prolonged single exciton lifetime

**Fig. 3:**
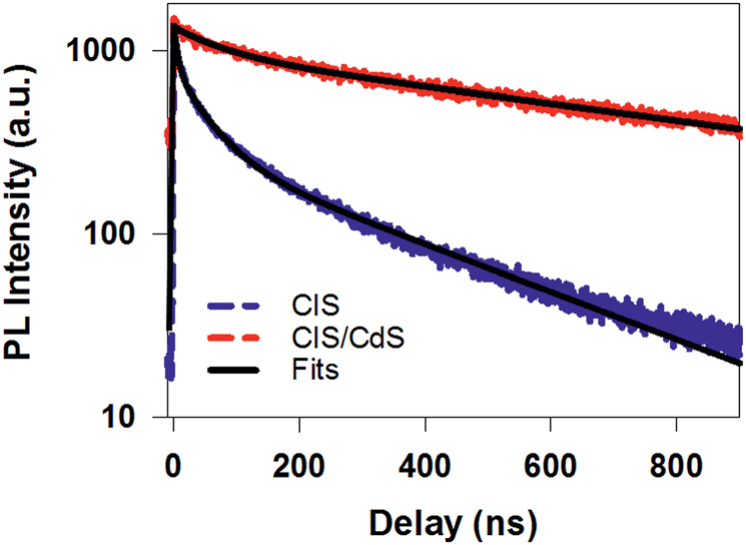
Time-resolved PL decay kinetics for CuInS_2_ core (blue dashed line) and CuInS_2_/CdS core/shell QDs (red dashed line) measured with 400 nm excitation. Their multi-exponential fits are shown in black solid lines and fitting parameters are listed in Table S1.†

It has been reported that the single exciton lifetime can be prolonged in quasi-type II core/shell QDs, such as CdSe/CdS and InP/CdS, as a result of both surface states passivation and reduced electron–hole overlap.^36,50^ Indeed, time-resolved PL decay measurements show that the PL lifetime was significantly lengthened from CIS QDs to CIS/CdS QDs (Fig. 3). The PL decay in CIS QDs can be fit by three exponential decay with time constants (and amplitude) of 5.8 ns (39.0%), 49.7 ns (39.6%), and 335 ns (21.4%), respectively (Table S1†). The slowest component agrees with reported radiative recombination time between CB edge electrons and V_Cu_ localized holes.^4,16^ Therefore, the two faster components can be attributed to surface trapping processes. The PL decay in CIS/CdS QDs can be well fit to a bi-exponential decay function with time constants (amplitudes) of 175 ns (49.8%) and 2200 ns (50.2%), respectively (Table S1†). Compared with CIS QDs, surface trapping is efficiently suppressed and single exciton radiative recombination time between CB edge electrons and V_Cu_ localized holes is significantly prolonged as a result of electron delocalization among the CIS core and CdS shell.

**Fig. 4:**
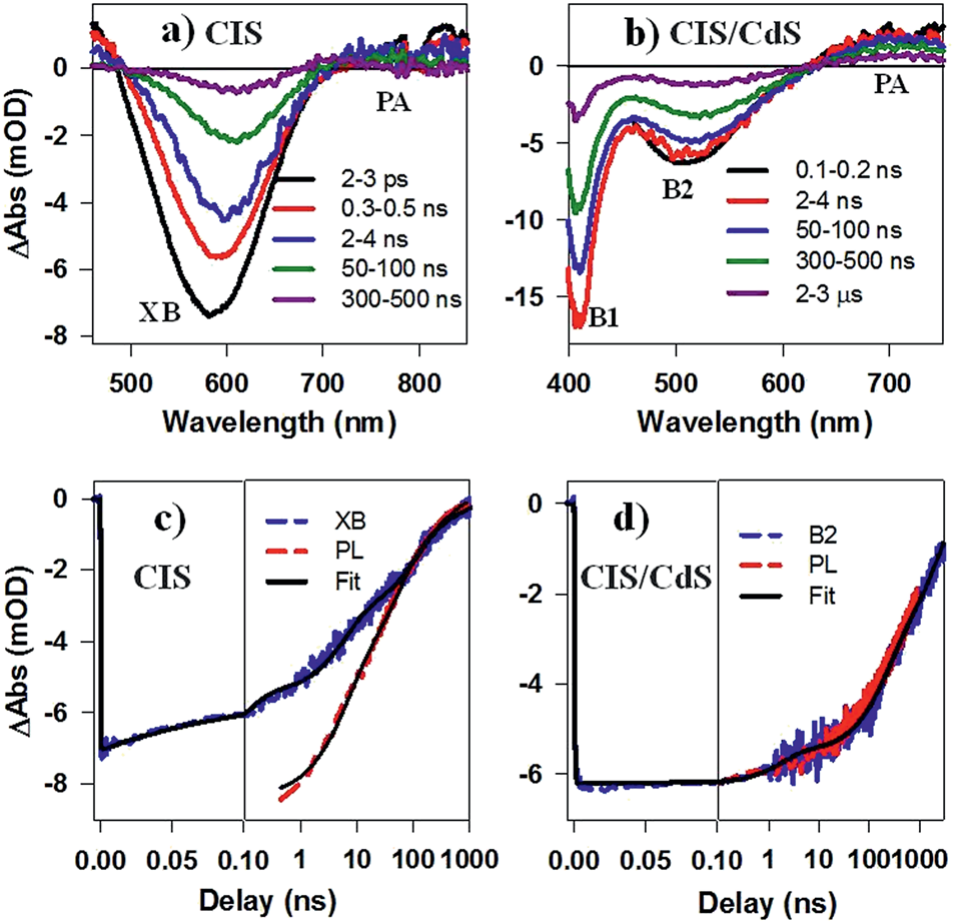
TA spectra of (a) CuInS_2_ core, and (b) CuInS_2_/CdS core/shell QDs at indicated delays after 400 nm excitation. Comparison of the kinetics of exciton bleach recovery (XB or B2, blue dashed line) and PL decay (red dashed lines) in (c) CuInS_2_ core, and (d) CuInS_2_/CdS core/shell QDs. Their multi-exponential fits are shown in black solid lines.

While PL decay can be caused by both electron or hole dynamics, TA can be used to investigate electron and/or hole dynamics separately.^53^ TA spectra of CIS QDs (Fig. 4a) measured with 400 nm excitation were dominated by an exciton bleach feature at ∼580 nm (XB) that can be attributed to state filling of the lowest energy CB electron level. TA spectra of CIS/CdS QDs (Fig. 4b) measured with 400 nm excitation showed B1 and B2 bleach features at ∼400 nm and 520 nm, respectively, consistent with those measured at 490 nm excitation (Fig. 2a). For both CIS and CIS/CdS QDs, there is an additional photoinduced absorption (PA) feature to the red of bleach features. The kinetics of PA is the same as the exciton bleach features in both samples (Fig. S4†). Similar broad and featureless PA signals have also been observed in many QDs, including CdSe,^54–56^ CdS,^36^ InP,^50,57^ and Cd_3_P_2_,^58^ and can be attributed to either holes or electrons or both. In Fig. 4c, we compared the kinetics of XB bleach recovery in CIS QDs with PL decay kinetics by normalizing them at long delay time (>100 ns), when the decay kinetics are the same and are dominated by recombination of CB electron with the trapped hole. The comparison showed that the PL decay was faster than exciton bleach in the time range of ∼1–100 ns. Since exciton bleach is determined by the CB electron, the fast components in its recovery kinetics suggest the presence of electron trapping processes. Furthermore, we attributed the much faster PL decay to hole trapping process in CIS QDs. There might exist hole trapping processes on faster time scale but not resolved due to the limited instrument response function (IRF ∼ 500 ps) of PL decay measurements. Our combined TA and PL decay measurements on CIS QDs revealed for the first time that both the CB edge electrons and the localized holes in V_cu_ are susceptible to surface trappings.

The B2 kinetics in CIS/CdS QDs measured with 400 nm excitation is shown in Fig. 4d. B1 and B2 showed the same kinetics and were the same as those measured at 490 nm excitation (Fig. S5†). Compared with XB kinetics in CIS QDs (half-life 9.8 ± 0.6 ns), B2 in CIS/CdS QDs is much longer lived (half-life 450 ± 20 ns), suggesting both effective passivation of electron traps due to shell growth and reduction of electron–hole recombination rates due to quasi-type II band alignment. Moreover, B2 bleach and PL decay kinetics in CIS/CdS QDs matched well with each other, suggesting that nearly all the hole trapping processes have been removed and the only decay channel for holes was through recombination with electrons, radiatively or nonradiatively. Thus, our results provided direct evidence for the passivation of both electron and hole traps in CIS/CdS QDs through the growth of CdS shell.

#### Suppressed Auger recombination

**Fig. 5:**
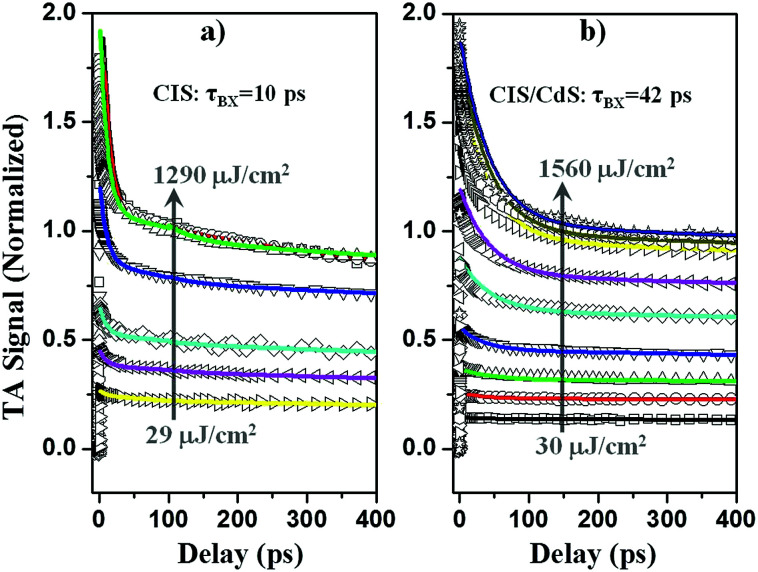
Normalized exciton bleach TA kinetics (open symbols) at different excitation intensities for (a) CuInS_2_ core, and (b) CuInS_2_/CdS core/shell QDs. The solid lines are fits to a multicarrier annihilation model described in the main text.

When more than one excitons are present in a QD, Auger recombination (AR), where an electron–hole pair recombines through excitation of the third particle (electron or hole), becomes the dominant pathway for multiple exciton annihilation.^21,59^ Previous studies suggest that AR rates can be effectively slowed down in quasi-type II core/shell QDs due to reduced electron–hole wavefunction overlap.^60,61^ Herein, we compared the multiple exciton dynamics of CIS/CdS QDs and CIS QDs to investigate the effect of quasi-type II band alignment on AR rate. The TA spectra of CIS and CIS/CdS QDs measured under different 400 nm excitation densities are plotted in Fig. S6 and S7,† respectively. It can be seen that there was an overlap of the PA signal with exciton bleach and the relative amplitudes of the PA signals increased with excitation densities. By assuming that PA amplitude was constant over the whole investigated spectral range, the contribution of PA signal to XB can be subtracted. As shown in Fig. 5, at low excitation densities, the XB signal was long-lived, consistent with the dominance of single exciton states; with increasing excitation densities, the amplitude of the fast decay component became larger, indicating the presence of multiple exciton states.

To determine multiple exciton AR rates, we first quantify the number of initially excited excitons in the QDs at different excitation powers by analyzing the power dependence of the XB signal amplitude. It is assumed that the probability of a QD absorbing *n* photons, *f*(*n*), follows the Poisson distribution: *f*(*n*) = *w*^*n*^e^−*w*^/*n*!, where *w*, the average number of excitons per QD, is proportional to the excitation density.^20,36,62,63^*P*(*n*, *t*) denotes the probability of finding QDs with *n* excitons at time *t*. At early delay time *t*_0_ ∼ 1 ps, prior to AR, *P*(*n*, *t*_0_) = *f*(*n*). At long delay time, *t*_L_ = 200 ps, when AR is completed and only single exciton states remain, *P*(1, *t*_L_) = 1 − *f*(0), and *P*(*n* > 1, *t*_L_) = 0; the transient XB signal amplitude, Δ*A*(*t*_L_), is proportional to the number of excited QDs: Δ*A*(*t*_L_) = *α*[1 − *f*(0)], where *α* is a scaling factor. It is convenient to define a normalized transient signal at *t*_L_:^36,58,63,64^

1 Δ*S*(*t*_L_) = Δ*A*(*t*_L_)/*α* = 1 − e^−*w*^

The scaling factor *α* can be determined by realizing that Δ*S*(*t*_L_) approaches 1 at high excitation intensities (*w* ≫ 1), when all QDs are excited. Similarly, normalized transient signals at all delay times, Δ*S*(*t*) = Δ*A*(*t*)/*α*, can also be obtained and are shown in Fig. 5. The initial normalized transient bleach signal at *t*_0_ is given by:^36,58,63,64^

2 Δ*S*(*t*_0_) = *P*(1, *t*_0_) + 2[1 − *P*(0, *t*_0_) − *P*(1, *t*_0_)] = 2 − (2 + *w*)e^−*w*^

where the factor of 2 arises from two-fold degeneracy of lowest electron level in CIS and CIS/CdS QDs. The normalized transient XB signals Δ*S*(*t*_0_) and Δ*S*(*t*_L_) as a function of excitation density are shown in Fig. S8.† Indeed, they can be fitted by eqn (1) and (2). From the fit, we obtain the initial average number of excitons *w* at any excitation density.

The normalized transient signal at any delay time is given by:^36,58,63,64^

3 Δ*S*(*t*) = *P*(1, *t*) + 2[1 − *P*(0, *t*) − *P*(1, *t*)]

The first and second terms represent the probability of single and multiple exciton (*n* > 1) states, respectively. Assuming that *n*-exciton state can only decay by sequential Auger recombination (to *n* − 1 exciton state with time constant *τ*_*n*_), the time-dependent distribution of multi-exciton states in QDs is described by a set of coupled rate equations:^36,59,63,65,66^4
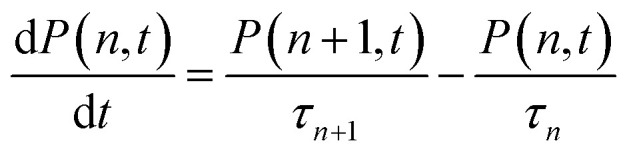


It has been demonstrated that the Auger recombination time of *n*-exciton states in QDs can be well described by a statistical scaling law: (*τ*_*n*_^−1^ = *n*^2^(*n* − 1)*τ*_2_^−1^/4), where *τ*_2_ is the bi-exciton AR lifetime constant.^36,59,63,67^ With the initial exciton population distribution obtained from fitting Fig. S8† and the bi-exciton lifetime as the only fitting parameter, the transient kinetics in Fig. 5 can be globally fitted by eqn (3) and (4), with emphasis on the lowest four powers. At higher powers, transient kinetics deviate from the model for the following reasons: first, although stirred, some of the QDs are likely photo-charged and show additional charging induced exciton decay channels;^68–70^ second, when *n* is large, Auger lifetime of *n*-exciton states in QDs might not obey the simple statistical scaling law.^71^ From the fit, we obtain bi-exciton lifetimes of 10 ± 2 ps and 42 ± 5 ps for CIS and CIS/CdS QDs, respectively. Another simple but not as rigorous way to extract bi-exciton lifetimes is to take the difference between kinetics at two lowest powers and attribute it to bi-exciton AR process.^59^ The bi-exciton lifetimes obtained from this simpler procedure are similar to the fitting results described above (Fig. S9†).

Therefore, we demonstrate that bi-exciton lifetime in CIS/CdS QDs is ∼4 times longer than in CIS QDs, which can be attributed to reduced wavefunction overlap between V_Cu_ localized holes in the CIS core and delocalized electrons among the CIS core and CdS shell. Suppressed AR in QDs is significant for their many applications. For instance, when using QDs to deliver multiple electrons or holes to an adsorbed catalyst molecule for solar fuel generation, interfacial charge transfer needs to compete with AR.^36,63,64,72,73^ Also, QDs are often unintentionally charged in devices such as QDSSCs, for which reason electron injection yield can be compromised by AR.^74^ In addition, AR is generally considered to be responsible for single QD PL blinking^75,76^ and gain decay in QD lasing processes.^77^ Therefore, suppressed AR in CIS/CdS QDs can improve their performances in these applications.

## Conclusions

In conclusion, we have presented a comprehensive ultrafast spectroscopic study of quasi-type II CIS/CdS core/shell QDs and demonstrated their improved light harvesting properties over CIS core only QDs. The CIS/CdS core/shell structures have a quasi-type II band alignment with a lowest energy CB electron level extending throughout the core and shell and a large VB offset. This band alignment is confirmed by the observation that the selective excitation of lowest CIS exciton band simultaneously generates long-lived CIS and CdS exciton bleaches. Through both ultrafast TA and time-resolved PL decay measurements, we show that electron and holes trapping states in CIS core are effectively passivated by the CdS shell and the single exciton state lifetime was prolonged to 450 ns as a result of reduced electron–hole overlap in quasi-type II CIS/CdS QDs. For the same reason, Auger recombination of multiple excitons was suppressed and the bi-exciton lifetime was extended to 42 ps in CIS/CdS QDs from 10 ps in CIS QDs. These findings suggest that quasi-type II CIS/CdS QDs are a promising material for photovoltaic and photocatalytic applications.

## Supplementary Material

SC-007-C5SC03715H-s001
