# Supplementary material for: Quasi-type II CuInS2/CdS core/shell quantum dots
Source: Chem Sci. 2015 Nov 12;7(2):1238–44. doi: 10.1039/c5sc03715h (PMC5975837; doi:10.1039/c5sc03715h)
Supplement: SC-007-C5SC03715H-s001 [file SC-007-C5SC03715H-s001.pdf]

Electronic Supplementary Material (ESI) for Chemical Science.
This journal is © The Royal Society of Chemistry 2015

Electronic Supplementary Information for:

Quasi-Type II $CuInS_2$/CdS Core/Shell Quantum Dots

Kaifeng Wu[a+], Guijie Liang [b+], Degui Kong [c], Jinquan Chen[a], Zheyuan Chen[a], Xinhe Shan [a], James R. McBride [d], and Tianquan Lian [a*]

[a] Department of Chemistry, Emory University,1515 Dickey Drive, NE, Atlanta, Georgia 30322,United States

[b] Hubei Key Laboratory of Low Dimensional Optoelectronic Materials and Devices, Hubei University of Arts and Science, Xiangyang 441053, Hubei Province, P. R. China

[c] College of Electronic Engineering, Heilongjiang University, Harbin 150080, P. R. China

[d] Department of Chemistry, The Vanderbilt Institute of Nanoscale Science and Engineering, Vanderbilt University, Nashville TN, 37235

[+] **These authors contribute equally to this work.**

***Corresponding author**: tlian@emory.edu

Content list:

S1. Sample synthesis and characterizations

S2. Transient absorption and time-resolved PL set-ups

S4. Kinetics fitting in CIS and CIS/CdS QDs

S3. Additional kinetics comparisons in CIS and CIS/CdS QDs

S5. Multi-exciton dynamics fitting in CIS and CIS/CdS QDs
S6. Charge separation and recombination in CIS and CIS/CdS QD-BQ Complexes

S1. Sample synthesis and characterizations

Synthesis of CIS and CIS/CdS QDs CIS QDs were synthesized following previously reported procedures.[1] In a typical synthesis, 0.292 g indium acetate, 0.190 g copper(I) iodide, and 5 mL 1-dodecanethiol (DDT) were mixed in a three-necked flask. The mixture was degassed for 30 min and then heated to 100 ˚C under argon purge until it turned into a clear solution. The temperature was then raised to 230 ˚C. During this process, the solution color changed from yellow to red, and finally black, as a result of increase in QD sizes. Aliquots of solution were taken out and their UV-vis absorption spectra were measured. The reaction was stopped after reaching desired absorption peak positions. The CIS QDs were precipitated out of the solution by adding ethanol. The precipitation was repeated for several times to remove excess ligands. The as-synthesized CIS QDs were used as core for CdS shell growth. CdS shell coating was achieved by successive ion layer adsorption and reaction (SILAR).[2] 0.1 M cadmium oleates in ODE and 0.1 M sulfur in ODE were used as precursor solutions. The precursors were injected into a flask with CIS QDs dissolved in ODE at 210 °C using an air-free syringe pump (with a rate of 1mL/1h). Due to core etching, it was difficult to calculate the amount of required precursors from the core size and shell layers. The reaction was stopped when desired absorption spectrum was obtained. The CIS/CdS QDs were precipitated out of the solution by adding ethanol.

The QDs were dispersed in heptane for spectroscopy studies. QD-BQ complexes were prepared by adding BQ powders to QD solution followed by sonication. All samples were filtered through 2 μm filters before transient absorption (TA) experiments to remove un-dissolved molecules and to minimize light scattering.

Sizes Distributions of QDs The sizes of CIS and CIS/CdS QDs were measured along their edges and the length distribution histograms are shown in Figure S1a and S1b, respectively. The average sizes (with standard deviations) of CIS and CIS/CdS QDs are 3.34(±0.95) nm and 4.25(±1.22) nm, respectively. STEM-EDS measurements were obtained using a Tecnai

Osiris operating at 200 kV with a probe current ~ 1.5 nA. EDS maps were generated using Bruker's Esprit software.

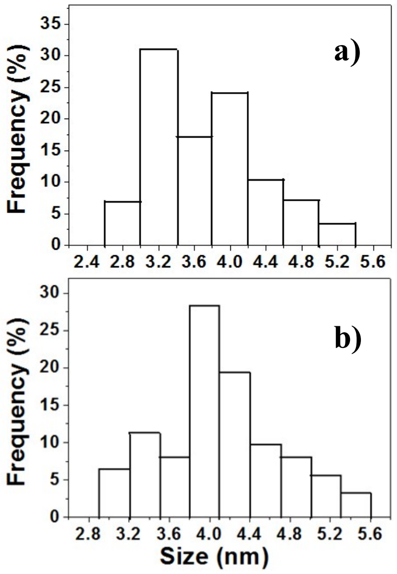

Figure S1. The size distribution histograms of (a) CIS, and (b) CIS/CdS QDs.

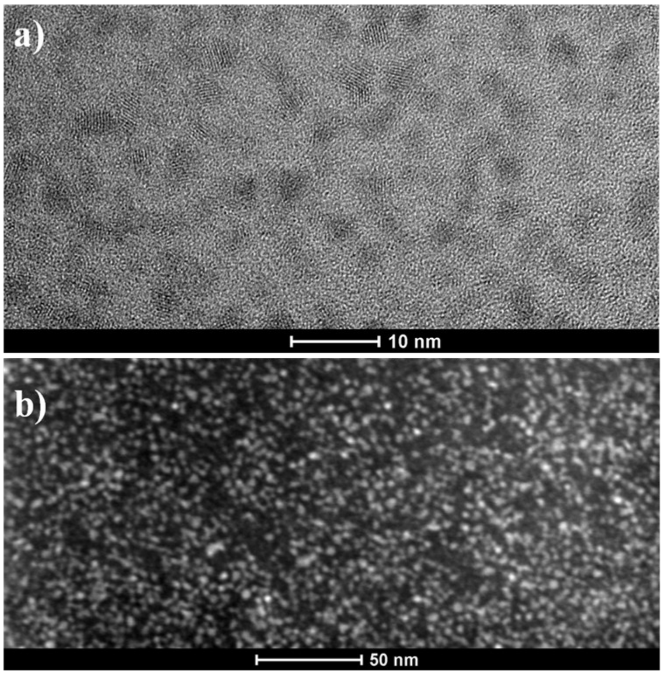

Figure S2. (a) High-resolution TEM (HR-TEM) and scanning TEM (STEM) images of CIS/CdS core/shell QDs.

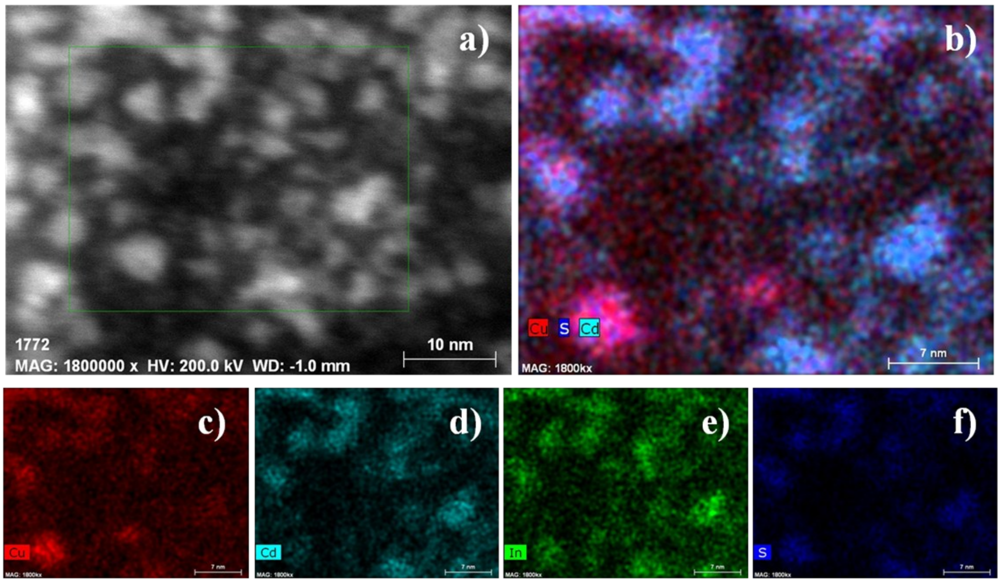

Figure S3. (a) An STEM image of CIS/CdS QDs used for EDX element mapping. (b) Overlaid Cu, S, and Cd element distributions. c)-f) Spatial distributions of Cu, Cd, In, and S.

S2. Transient absorption and time-resolved PL set-ups

Visible femtosecond transient absorption spectrometer. A regeneratively amplified Ti:sapphire laser system (Coherent Legend, 800 nm, 150 fs, 2.3 mJ/pulse, and 1 kHz repetition rate) and Helios (Ultrafast Systems LLC) spectrometers were used for the transient absorption measurements. The 800 nm output pulse from the regenerative amplifier was split in two parts with a 50% beam-splitter. One part was used to pump a Coherent Opera Optical Parametric Amplifier (OPA) which generates the signal and idler beams. The former was used to generate the 490 nm excitation beam by sum frequency mixing with the 800 nm fundamental beam in a BBO crystal. 400 nm pump beam was made by frequency-doubling of one part of 800 nm beam in the BBO crystal. A series of neutral-density filters was used to adjust the power of the pump beam. The pump beam was focused at the sample with a beam waist of about 360 μm. A white light continuum (WLC) from 420 to 800 nm was generated by attenuating and focusing ~10 μJ of the 800 nm pulse into a sapphire window. The WLC was split into a probe and reference beam. The probe beam was focused with an Al parabolic reflector onto the sample (with a beam waist of 150 μm at the sample). Both the reference and probe beams were focused into a fiber optics-coupled multichannel spectrometer with complementary metal-oxide-semiconductor (CMOS) sensors and detected at a frequency of 1

kHz. The intensity of the reference beam was used to correct the pulse-to-pulse fluctuation of the white-light continuum. The pump beam was chopped by a synchronized chopper to 500 Hz; the probe intensities of the pumped and unpumped sample were compared to calculate the pump induced absorbance change. The delay time between the pump and probe pulses was controlled by a motorized delay stage. The instrument response function (IRF) of this system was determined to be ~150 fs by measuring solvent responses under the same experimental conditions (with the exception of a higher excitation power). The sample was held in an 1 mm quartz curvette and stirred constantly by a magnetic stirrer during the measurements.

Visible Nanosecond Transient Absorption Nanosecond TA was performed with the EOS spectrometer (Ultrafast Systems LLC). The pump beams at 400 nm and 490 nm were generated in the same way as the femtosecond TA experiments described above. The white light continuum (380-1700 nm, 0.5 ns pulse width, 20 kHz repetition rate) used here was generated by focusing a Nd:YAG laser into a photonic crystal fiber. The delay time between the pump and probe beam was controlled by a digital delay generator (CNT-90, Pendulum Instruments). The probe and reference beams were detected with the same multichannel spectrometers used in the femtosecond TA experiments. The IRF of this system was measured to be ~280 ps. The data from femtosecond and nanosecond transient spectrometers overlap at delay time windows from 0.5 to 1 ns. To connect spectra and kinetics from these spectrometers, we scale the amplitudes of the nanosecond data by a scaling factor to agree with the femtosecond results in the overlapping delay time windows.

Time-Resolved Fluorescence Lifetime Measurements. Time-correlated single photon counting (TCSPC) method was used to measure the fluorescence decay of the QDs. Samples were hold in a 1cm cuvette and measured at the right angle geometry. The output pulses centered at 800 nm (∼100 fs, 80 MHz) from a mode-locked Ti:Sapphire laser (Tsunami oscillator pumped by a 10 W Millennia Pro, Spectra-Physics) were passed through a pulse picker (Conoptics, USA) to reduce the repetition rate by a factor of 80 and then frequency-doubled in a BBO crystal to generate pump pulses at 400 nm and used to excite QD samples. The emissions from QDs were detected by a microchannel-plate photomultiplier tube

(Hamamatsu R3809U-51), the output of which was amplified and analyzed by a TCSPC board (Becker & Hickel SPC 600). The IRF of this system was determined to be ~500 ps.

S3. Kinetics fitting in CIS and CIS/CdS QDs

Time-resolved PL decay kinetics of CIS and CIS/CdS QDs are fitting with the following equation:

$$PL(t) = A \cdot [\sum_{i=1}^{3} a_i \cdot e^{-t/\tau_i} - e^{-t/\tau_f}] \quad \text{(S1)},$$

where A is the initial signal size, a_i and τ_i are the amplitude and time constant of multi-exponentials, and τ_f is the signal formation time constant. Similarly, the transient XB kinetics can be also fitted with equation S1. These fitting parameters and errors are listed in Table S1.

Table S1. Fitting Parameters for PL and XB Kinetics

		τ_f/ps	τ_1/ns (a_1)	τ_2/ns (a_2)	τ_3 /ns (a_3)	$\tau_{1/2}$ /ns
PL	CIS	<<500	5.8±0.1 (39.0±0.4%)	49.7±0.6 (39.6±0.3%)	335±3 (21.4±0.2%)	18±2
	CIS/CdS	<<500	175±3 (49.8±0.7%)	2200±50 (50.2±0.8%)	NA	660±20
XB	CIS	0.16±0.06	0.20±0.02 (26.2±0.6%)	11.0±0.4 (35.0±0.5%)	320±10 (38.8±0.4%)	9.8±0.6
	CIS/CdS	0.43±0.08	1.5±0.2 (11.9±0.5%)	207±8 (35.6±0.9%)	2356±70 (52.5±0.9%)	450±20

S4. Additional kinetics comparisons in CIS and CIS/CdS QDs

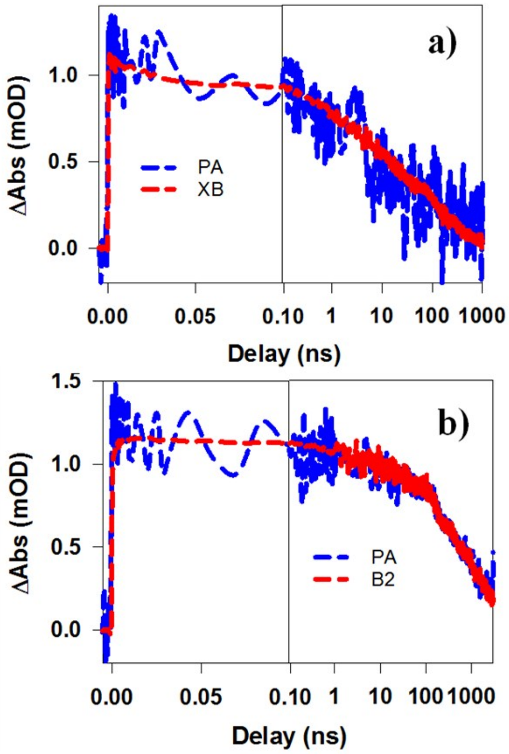

Figure S4. Comparisons between XB (or B2) (red dashed lines) and PA (blue dashed lines) kinetic in (a) CIS, and (b) CIS/CdS QDs.

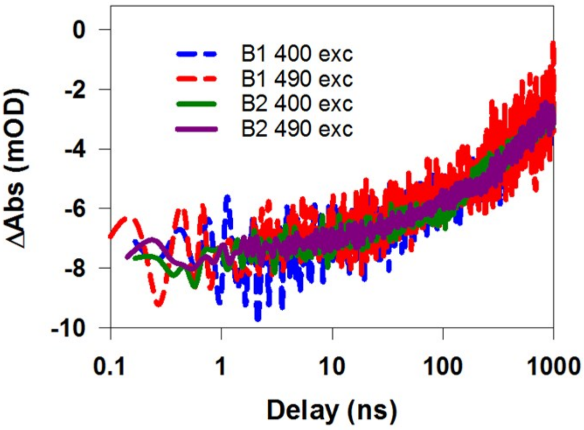

Figure S5. B1 and B2 kinetics in CIS/CdS QDs under both 400 nm and 490 nm excitations. They all agree with each other.

S5. Multi-exciton dynamics fitting in CIS and CIS/CdS QDs

Figure S6 and S7 show the excitation (400 nm) density dependent TA spectra of CIS and CIS/CdS QDs, respectively. The PA signal to the red of XB feature increases strongly with excitation density. Although we show in Figure S5 that in both CIS and CIS/CdS QDs,

PA and XB have the same kinetics under low excitation density, they have different saturation behavior at high excitation density and therefore their kinetics can be different. To account for this, the kinetics of PA were subtracted from those of XB to obtain real XB kinetics shown in Figure 5.

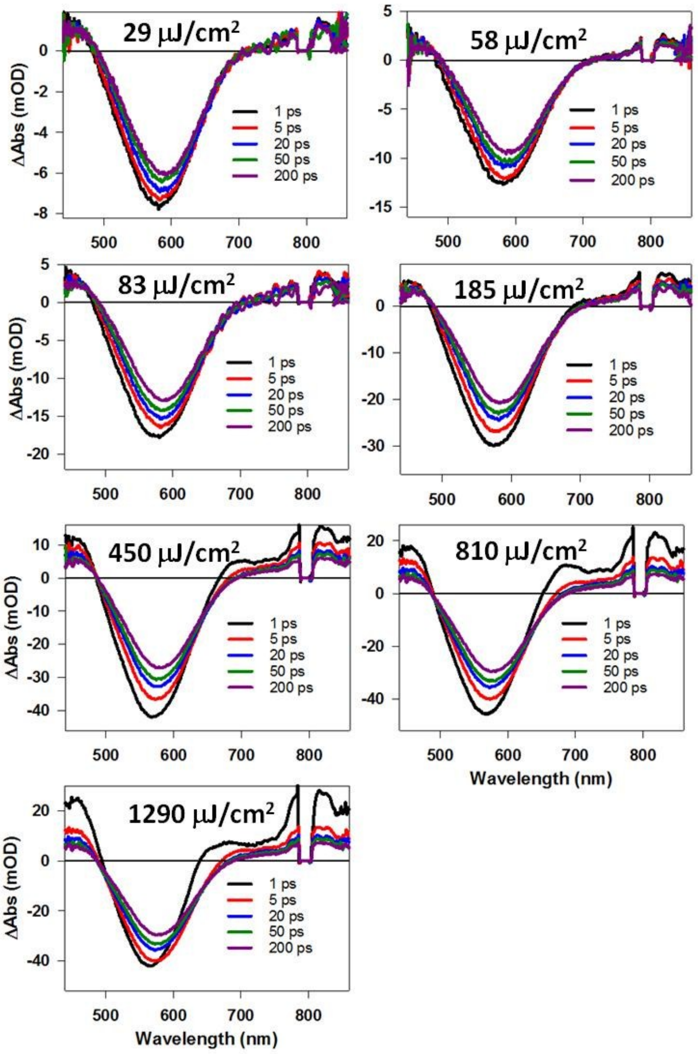

Figure S6. TA spectra of CIS QDs at different excitation densities.

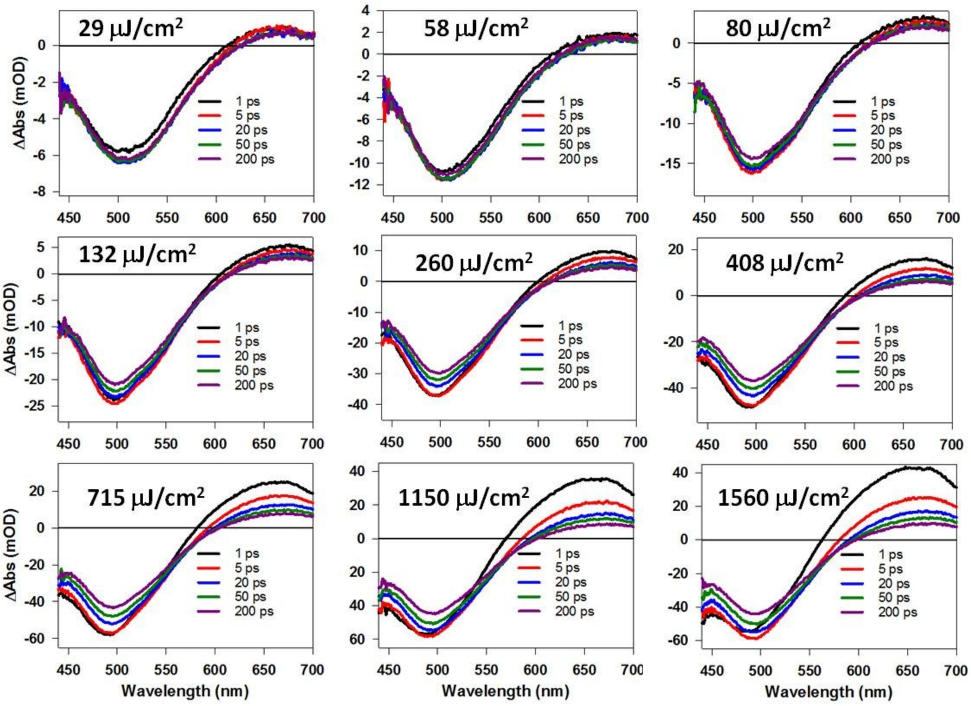

Figure S7. TA spectra of CIS/CdS QDs at different excitation densities.

We fit the normalized transient XB signals $\Delta S(t_0=1\text{ ps})$ and $\Delta S(t_L=200\text{ ps})$ in CIS and CIS/CdS QDs as a function of excitation densities according to equation 1 and 2 in the main text. Since the parameter *w,* the initial average number of excitons per QD, is proportional to the excitation density (*I*): *w=CI, C* is the only fitting parameter. *C* is proportional to the absorption cross section of QDs and it is different for CIS and CIS/CdS QDs. As shown in Figure S8, the experimental data can be well fitted with this model and from the fitting we obtain *w*, the initial average number of excitons per QD. These *w* values will be used to fitted the normalized XB kinetics in Figure 5 according to equation 3 and 4.

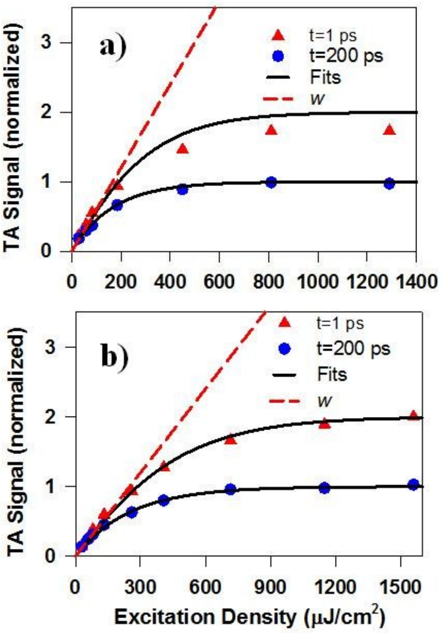

Figure S8. Normalized transient XB signals ΔS(t_0=1 ps) (red triangles) and ΔS(t_L=200 ps) (blue circles) in (a) CIS, and (b) CIS/CdS QDs as a function of excitation density. The black solid lines are fits according to the model described in the main text. The red dashed lines are fitted average exciton numbers per QD as a function of excitation density.

We also tried extracting bi-exciton lifetimes by taking the difference between kinetics at two lowest powers. The differential kinetics are shown in Figure S9a and S9b for CIS and CIS/CdS QDs, respectively. We attribute them to bi-exciton Auger recombination process.[3] Fitting them generates bi-exciton lifetimes of 9±3 ps and 46±6 ps for CIS and CIS/CdS QDs, respectively. These results are consistent with those obtained from excitation density dependent kinetics fitting in Figure 5.

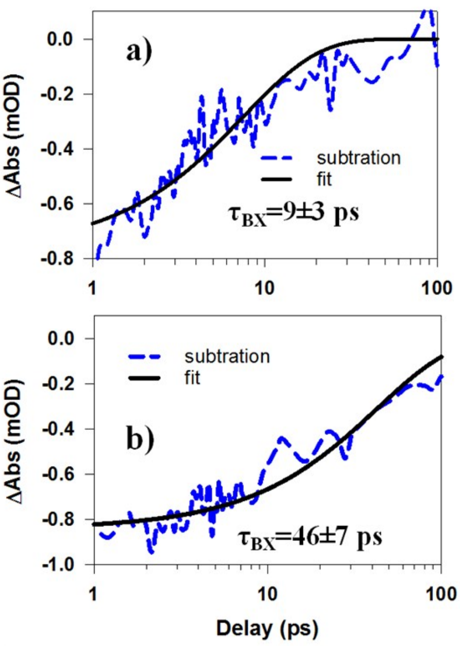

Figure S9. Bi-exciton Auger recombination process in (a) CIS, and (b) CIS/CdS QDs obtained by kinetics subtraction (blue dashed lines). The black solid lines are single-exponential fits to them.

S6. Charge separation and recombination in CIS and CIS/CdS QD-BQ Complexes

We measured charge separation and recombination kinetics in CIS and CIS/CdS QD-benzoquinone (BQ) donor-acceptor complexes. This experiment can also assign TA features to specific (electron and/or hole) transitions. BQ is chosen because it has a suitable redox potential and the reduced BQ anion has negligible absorption in the visible region which would otherwise overlap with QD signals.[4,5] Figure S10a shows the TA spectra of CIS QD-BQ complexes at different delays after 400 nm excitation. Both XB and PA features show faster decay than free CIS QDs, as a result of electron transfer to BQ, and their kinetics are the same within ~500 ps (Figure S11a). At ~1 ns, PA completely disappears and therefore we assign it to induced absorption of electrons, similar to InP QDs.[6] In contrast, XB feature did not completely decay to zero at ~ 1ns, which can be attributed to charge separation (CS) induced Stark effect signal (see spectrum in Figure S11b), similar to previous observations in many QD-acceptor systems.[6-9] Therefore, we can use PA to extract charge separation kinetics and CS to monitor charge recombination process in CIS QD-BQ complexes.

TA spectra of CIS/CdS QD-BQ complexes at different delays after 400 nm excitation are shown in Figure S10b. Ultrafast B2 and PA decay was observed as a result of electron transfer to BQ. However, the PA signal did not completely recover in ~ 1ns. Therefore, both electrons (~70%) and holes (~30%) contributed to PA feature in CIS/CdS QDs. In addition, CS signal was not clearly observed and was likely obscured by the overlapping PA feature. However since the remaining PA feature at long delay times corresponds to holes, it can be used to monitor charge recombination process. We can subtract PA kinetics from B2 kinetics to extract charge separation kinetics.

The charge separation kinetics in CIS-BQ and CIS/CdS-BQ complexes are compared in Figure S10c and they are fitted to eq. S1. The fitting parameters were listed in Table S2. Since BQ/QD molar ratios were controlled to be the same for these QDs, the kinetics can be directly compared to determine the difference in interfacial electron transfer rates. Surprisingly, the observed electron transfer rate from CIS/CdS core/shell QDs (half-life 7.0±0.5 ps) was faster than CIS QDs (half-life 33±7 ps). We attribute it to etched core size in the CIS/CdS QDs during the growth process, which leads to stronger quantum confinement and therefore larger driving force for electron transfer.[10] Charge separation yield in QD-BQ complexes was estimated according to:[11] $\Phi_{CS} = (\tau_{QD} - \tau_{QD-BQ})/\tau_{QD}$, where τ_{QD} and τ_{QD-BQ} are the electron half-lives in free QDs and QD-BQ complexes, respectively. The calculated yields for CIS QD-BQ and CIS/CdS-BQ were 99.7% and 100%, respectively. The higher charge separation yield in the latter can be attributed to prolonged electron lifetime in quasi-type II QDs and faster electron transfer rates.

Charge recombination kinetics in CIS-BQ and CIS/CdS-BQ complexes are compared in Figure S10d and they are also fitted to eq. S1. The fitting parameters were also listed in Table S2. The charge separated state CIS/CdS$^+$-BQ$^-$ (half-life 85±10 ns) was 53 times longer lived than CIS $^+$-BQ$^-$ (half-life 1.6±0.3 ns), as a result of quasi-type II band alignment. According to Scheme 1, the CdS shell acts as an efficient barrier (~1 eV) to prevent recombination between the V_{cu} localized hole in the core and electron in the LUMO of BQ.

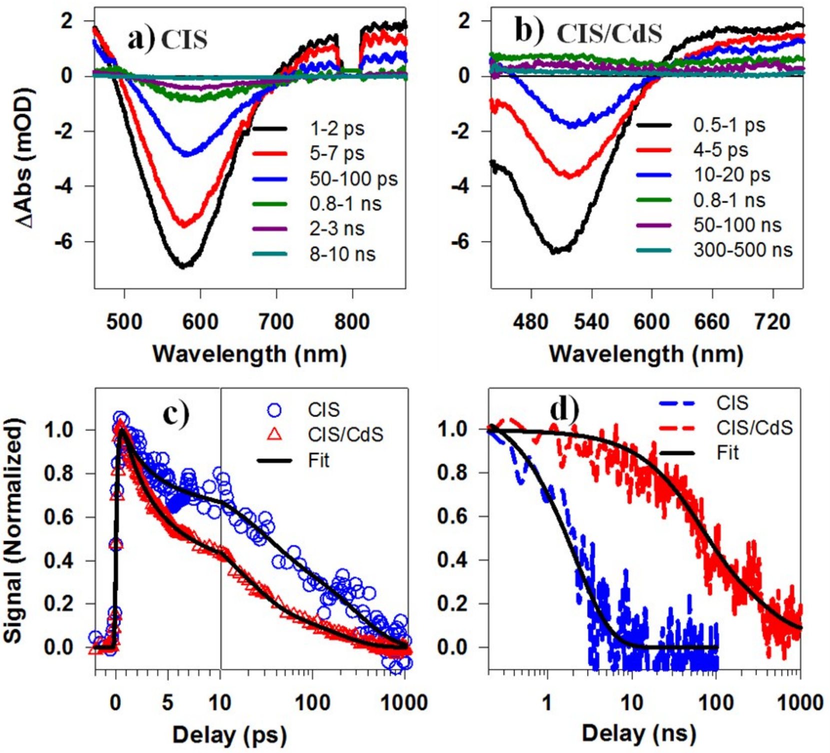

Figure S10. TA spectra a) $CuInS_2$ core, and b) $CuInS_2$/CdS core/shell QDs in the presence of benzoquinone (BQ) electron acceptors at different delays after 400 nm excitation. c) Normalized charge separation kinetics for $CuInS_2$-BQ (blue circles) and $CuInS_2$/CdS-BQ (red triangles). d) Normalized charge recombination kinetics for $CuInS_2^+$-BQ^- (blue dashed line) and $CuInS_2/CdS^+$-BQ^- (red dashed line). The black solid lines are multi-exponential fits to these kinetics.

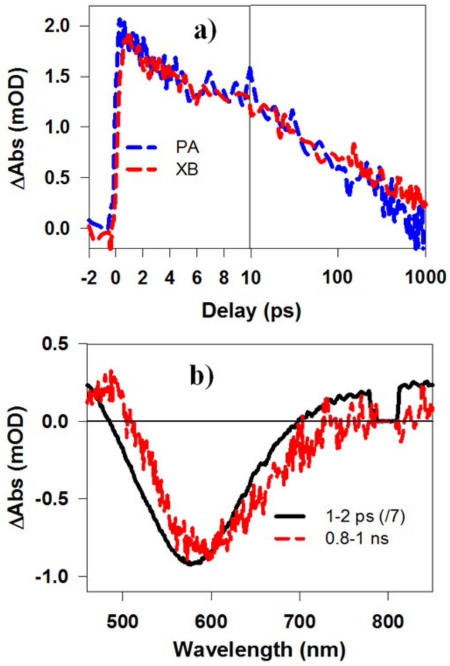

Figure S11. a) Comparison of PA (blue dashed line) and XB (red dashed line) kinetics in $CuInS_2$ QD-BQ complexes. b) Zoom-in of charge separated state signal at 0.8-1 ns (red dashed line) in $CuInS_2$ QD-BQ complexes. The state filling signal at 1-2 ps is reduced by a factor of 7 for comparison.

Table S2. Fitting Parameters for CS and CR Kinetics

		τ_1/ps (a_1)	τ_2/ps (a_2)	τ_3 /ps (a_3)	$\tau_{1/2}$ /ps	Φ_{CS}
CS	CIS	2.5±0.9 (26.0±5.0%)	26.1±8.0 (30.9±5.5%)	290±38 (43.1±4.7%)	33±7	99.7%
	CIS/CdS	2.8±0.1 (46.2±1.7%)	17.9±1.6 (35.8±1.6%)	162±11 (17.9±1.2%)	7.0±0.5	100%
CR		τ_1/ns (a_1)	τ_2/ns (a_2)	τ_3 /ns (a_3)	$\tau_{1/2}$ /ns	
	CIS	0.55±0.20 (14.7±4.0%)	2.1±0.6 (85.3±6.5%)	NA	1.6±0.3	
	CIS/CdS	56±8 (53.8±7.4%)	318±99 (38.9±6.0%)	>>1000 (7.3±2.6%)	85±10	

References for SI:

(1) Li, L.; Pandey, A.; Werder, D. J.; Khanal, B. P.; Pietryga, J. M.; Klimov, V. I.: Efficient Synthesis of Highly Luminescent Copper Indium Sulfide-Based Core/Shell Nanocrystals with Surprisingly Long-Lived Emission. *J. Am. Chem. Soc.* **2011**, *133*, 1176-1179.

(2) Li, J. J.; Wang, Y. A.; Guo, W. Z.; Keay, J. C.; Mishima, T. D.; Johnson, M. B.; Peng, X. G.: Large-scale synthesis of nearly monodisperse CdSe/CdS core/shell nanocrystals using air-stable reagents via successive ion layer adsorption and reaction. *J. Am. Chem. Soc.* **2003**, *125*, 12567-12575.

(3) Klimov, V. I.; Mikhailovsky, A. A.; McBranch, D. W.; Leatherdale, C. A.; Bawendi, M. G.: Quantization of multiparticle Auger rates in semiconductor quantum dots. *Science* **2000**, *287*, 1011-1013.

(4) McArthur, E. A.; Morris-Cohen, A. J.; Knowles, K. E.; Weiss, E. A.: Charge Carrier Resolved Relaxation of the First Excitonic State in CdSe Quantum Dots Probed with Near-Infrared Transient Absorption Spectroscopy†. *J. Phys. Chem. B* **2010**, *114*, 14514-14520.

(5) Burda, C.; Green, T. C.; Link, S.; El-Sayed, M. A.: Electron Shuttling Across the Interface of CdSe Nanoparticles Monitored by Femtosecond Laser Spectroscopy. *J. Phys. Chem. B* **1999**, *103*, 1783-1788.

(6) Wu, K.; Song, N.; Liu, Z.; Zhu, H.; Rodríguez-Córdoba, W.; Lian, T.: Interfacial Charge Separation and Recombination in InP and Quasi-Type II InP/CdS Core/Shell Quantum Dot-Molecular Acceptor Complexes. *J. Phys. Chem. A* **2013**, *117*, 7561-7570.

(7) Zhu, H. M.; Song, N. H.; Lian, T. Q.: Controlling Charge Separation and Recombination Rates in CdSe/ZnS Type I Core-Shell Quantum Dots by Shell Thicknesses. *J. Am. Chem. Soc.* **2010**, *132*, 15038-15045.

(8) Zhu, H.; Song, N.; Lian, T.: Wave Function Engineering for Ultrafast Charge Separation and Slow Charge Recombination in Type II Core/Shell Quantum Dots. *J. Am. Chem. Soc.* **2011**, *133*, 8762-8771.

(9) Wu, K.; Liu, Z.; Zhu, H.; Lian, T.: Exciton Annihilation and Dissociation Dynamics in Group II–V Cd3P2 Quantum Dots. *J. Phys. Chem. A* **2013**, *117*, 6362-6372.

(10) Zhu, H.; Yang, Y.; Hyeon-Deuk, K.; Califano, M.; Song, N.; Wang, Y.; Zhang, W.; Prezhdo, O. V.; Lian, T.: Auger-Assisted Electron Transfer from Photoexcited Semiconductor Quantum Dots. *Nano Lett.* **2014**, *14*, 1263-1269.

(11) Wu, K.; Chen, Z.; Lv, H.; Zhu, H.; Hill, C. L.; Lian, T.: Hole Removal Rate Limits Photo-driven H2 Generation Efficiency in CdS-Pt and CdSe/CdS-Pt Semiconductor Nanorod-metal tip Heterostructures. *J. Am. Chem. Soc.* **2014**, *136*, 7708-7716.
